# Successful live birth of a healthy infant by preimplantation genetic testing for aneuploidy in a couple with recurrent spontaneous abortion: a case report

**DOI:** 10.3389/frph.2025.1627160

**Published:** 2025-08-08

**Authors:** Yuqin Zhu, Zhuoyao Mai, Ruiqi Li, Zaowen Liao, Nengyong Ouyang, Hui Chen, Haijing Zhao

**Affiliations:** ^1^ Reproductive Medicine Center, Sun Yat-sen Memorial Hospital of Sun Yat-sen University, Guangzhou, Guangdong, China; ^2^ Reproductive Medicine Center, Shenshan Central Hospital, Sun Yat-sen Memorial Hospital of Sun Yat-sen University, Shanwei, Guangdong, China; ^3^ Guangdong Provincial Clinical Research Center for Obstetrical and Gynecological Diseases, Guangzhou, Guangdong, China; ^4^ Postdoctoral Research Station of Clinical Medicine, Xinjiang Medical University, Ürümqi, Xinjiang, China; ^5^ Reproductive Medicine Center, Hainan Kapok Hospital, Qionghai, Hainan, China; ^6^ Reproductive Medicine Center, The First People's Hospital of Kashgar, Kashgar, China

**Keywords:** recurrent spontaneous abortion, embryonic chromosomal factor, maternal factor, sperm DNA integrity, external factor

## Abstract

Recurrent spontaneous abortion (RSA), characterized by the occurrence of three or more pregnancy losses prior to the 28th week of gestation, stands as a formidable challenge in the realm of assisted reproductive medicine, presenting significant difficulties in both clinical diagnosis and treatment. In recent years, there has been a discernible upward trend in the incidence of RSA, and its etiology is multifaceted and intricate. Pinpointing the root causes of RSA remains an arduous task that urgently demands resolution within the field of reproductive medicine. Through a comprehensive and in-depth analysis of the RSA-related case report, embryonic chromosomal abnormalities were identified as the highest-risk factor. Utilizing preimplantation genetic testing for aneuploidy (PGT-A) technology, an euploid blastocyst was selected for transfer, which led to successful pregnancy and healthy birth. This case holds significant reference value for the clinical treatment of RSA.

## Introduction

Recurrent spontaneous abortion (RSA) is a distressing condition affecting approximately 1% of women attempting pregnancy (1). The definition of RSA refers to the occurrence of three or more pregnancy losses before 28 weeks of gestation in pregnancies with the same sexual partner (1, 2). However, most experts emphasize that two pregnancy losses should warrant attention. Clinically, the etiologies of three and two miscarriages are similar, and their subsequent recurrence rates are also comparable (1). Moreover, the damage incurred by each abortion will reduce the possibility of subsequent pregnancy. The etiology of RSA is complex and multifaceted, with approximately 50% of cases remaining unexplained—referred to as recurrent spontaneous abortion (URSA) (3).

In this study, couples with a 1-year history of secondary infertility sought treatment at the Reproductive Medicine Center of Sun Yat-sen Memorial Hospital. The woman had undergone three cleavage-stage embryo transfers, unfortunately, all of these attempts ended in early spontaneous abortions. Considering the embryonic chromosomal abnormalities observed in the second cycle, preimplantation genetic testing for aneuploidy (PGT-A) was performed. During the subsequent frozen embryo transfer (FET) cycle, a chromosomally normal blastocyst was transferred. The woman then successfully achieved clinical pregnancy and delivered a healthy live infant following regular follow-up.

Based on this distinctive case, we comprehensively detailed the couple's information, relevant examinations, clinical treatments and outcomes. Moreover, we provided an in-depth review of the relevant factors contributing to her RSA.

## Case description

### Female information

The patient is a 34-year-old woman with a body mass index (BMI) of 22 kg/m^2^. Her menstrual cycle is usually 6–7 days every 27–28 days, with a moderate amount of bleeding and no dysmenorrhea. In January 2017, she underwent surgical treatment for intramural fibroids in our hospital. Hysteroscopy showed endometrial polypoid hyperplasia, and electroresection was performed. Laparotomy revealed extensive pelvic adhesions. Both fallopian tubes and ovaries were tightly adhered to the pelvic cavity. The fimbria of the right fallopian tube was atretic, and the left one was incompletely atretic with the disappearance of the fimbrial structure. Intraoperative hydrotubation showed that the left fallopian tube was poorly filled, and the right one was blocked. Therefore, myomectomy, pelvic adhesiolysis, and bilateral salpingectomy were performed.

In 1995, she underwent surgical repair for congenital heart disease (ventricular septal defect). In January 2017, echocardiography showed mild mitral and tricuspid regurgitation. She has a history of alcohol allergy. She had one induced abortion due to an unplanned pregnancy.

Physical and gynecological examinations were normal. Cervical cell and HPV tests showed no intraepithelial lesions or malignancies. Her chromosomal karyotype was 46, XX. In July 2017, the basic endocrine test showed: follicle stimulating hormone (FSH)/luteinizing hormone (LH) = 8.6/5.15, estradiol (E_2_) < 20 ng/L, and CA125 = 16.1 U/ml.

### Male information

The male partner of that woman is 43 years old with a BMI of 25.3 kg/m^2^. He has normal erectile function, normal duration of sexual activity, and normal ejaculation function. In July 2017, the sperm analysis showed that the total sperm count (TSC) is 14 million/ml, progressive motility (PR) is 25%, and the normal morphology rate is 1%. The scrotal ultrasound showed no obvious abnormalities. There is no special information in his past medical history, personal history and reproductive history.

There is a family history of thalassemia. The male partner is diagnosed with male infertility, mild oligospermia, mild asthenospermia, and mild α-thalassemia. His chromosomal karyotype was 46, XY.

### History of treatment

The long luteal phase protocol was adopted in the first cycle. Triptorelin (GnRHa, Dabijia, Huiling Company, 3.75 mg) at a dose of 1.25 mg was administered subcutaneously for down-regulation. Fourteen days later, recombinant human follicle-stimulating hormone (r-hFSH, Gonafen, Merck Serrano, 75 IU) was initiated at a subcutaneous injection dose of 150 U per day. After 3 days of administration, hormone levels were measured: E_2_ was 82 pg/ml and LH was 1.14 mIU/ml. Considering the follicular development, the dose of r-hFSH was increased to 187.5 U per day for subcutaneous injection and administered for four consecutive days. Hormone levels were re-measured: E_2_ increased to 738 pg/ml and LH decreased to 0.93 mIU/ml. Subsequently, the dose of r-hFSH was adjusted to 150 U per day for subcutaneous injection, and highly purified human menopausal gonadotropin (uHMG, HemeiQi, Huiling Company, 150 U) at a dose of 75 U per day was added for intramuscular injection. After 3 days of combined administration, hormone levels were re-checked: E_2_ reached 3,131 pg/ml and LH was 1.55 mIU/ml. The administration of 150 U of r-hFSH subcutaneously and 75 U of uHMG intramuscularly was continued for one more day. Then, recombinant human chorionic gonadotropin (rHCG, Aize, Merck Serrano, 250 ug) at a dose of 250 μg was injected subcutaneously for triggering ovulation. Oocyte retrieval was performed 36 h after triggering under the monitoring of transvaginal ultrasound. In the fresh cycle, two cleavage-stage embryos were transferred, but early spontaneous abortion (7 weeks) occurred, and chorionic villus tissue testing was not performed.

Ultra-long GnRH agonist protocol was adopted in the second cycle 1 year later. Triptorelin (GnRHa, Dapagin, Yipu Sheng Company, 3.75 mg) is administered subcutaneously at a dose of 1.25 mg each time. A total of two down-regulation injections are given, with an interval of 15 days between the two administrations. Fourteen days after the last down-regulation, recombinant human follicle-stimulating hormone (r-hFSH, Pulikon, MSD, 300 IU) is used to initiate ovulation induction at a subcutaneous injection dose of 175 U per day for five consecutive days. At this time, the hormone levels are E_2_ level of 495 pg/ml and LH level of 0.79 mIU/ml. The dosage of r-hFSH is reduced to 100 U per day for subcutaneous injection. Meanwhile, uHMG 75 U is added for intramuscular injection per day, and this regimen is used for 3 days. At this time, the hormone levels change to E_2_ level of 3,937 pg/ml and LH level of 3.10 mIU/ml. Continue the subcutaneous injection of r-hFSH at 100 U and the intramuscular injection of uHMG at 75 U per day for one more day. Then, human chorionic gonadotropin (HCG, Zhuhai Lizhu Group, 2,000 U) at a dose of 10,000 U is administered intramuscularly for triggering. Oocyte retrieval is performed 36 h after triggering under the monitoring of transvaginal ultrasound. In the fresh cycle, two cleavage-stage embryos were transferred, but early spontaneous abortion (12 weeks) still occurred. The chorionic villus tissue test results showed: 45, X.

After 7 months, GnRH antagonist was adopted in the third cycle. Ovulation induction was initiated with subcutaneous injection of 200 U of r-hFSH per day for 5 days. After that, the hormone levels were measured, showing an E_2_ level of 902 pg/ml and an LH level of 4.79 mIU/ml. The subcutaneous injection of 200 U of r-hFSH per day was continued. Additionally, gonadotropin-releasing hormone antagonist ganerik acetate (GnRH-A, Eucalyptus, Mercado, 0.25 mg) was subcutaneously injected at a dose of 0.25 mg per day for 3 days. Then, the hormone levels were re-measured, with an E_2_ level of 2,688 pg/ml and an LH level of 2.26 mIU/ml. The subcutaneous injection of 200 U of r-hFSH per day was continued for one more day. Then, 10,000 U of HCG was intramuscularly injected, and 0.1 mg of triptorelin (GnRHa, Dafirin, Iproxen, 0.1 mg) was subcutaneously injected for triggering. Oocyte retrieval was performed under transvaginal ultrasound monitoring 36 h after triggering. In the fresh cycle, one cleavage-stage embryo was transferred, unfortunately, early spontaneous abortion recurred (9 weeks), and chorionic villus tissue testing was not performed.

Ovulation induction protocol was adopted in FET cycle 2 years later. Intramuscular injection of urinary gonadotropin (HMG, Zhuhai Lizhu Group, 75 IU) was used for ovulation induction treatment at a dosage of 75 U per day via intramuscular injection. After four consecutive days of initial administration, follicular monitoring was carried out, revealing that the largest follicle on the left side had a diameter of 8.5 mm, and the largest follicle on the right side also measured 8.5 mm in diameter. Based on these monitoring results, HMG treatment was continued for another 5 days. Subsequently, hormone level detection and follicular monitoring were performed. The hormone values were E_2_ level of 244 pg/ml, LH level of 85.15 mIU/ml, and P level of 0.49 ng/ml. Follicular monitoring showed that the diameter of the largest follicle on the left side increased to 18.0 mm, while the largest follicle on the right side remained at 8.5 mm. Meanwhile, the thickness of the endometrium was 11.2 mm, with a classification of type A. One day after the above monitoring results, luteal support treatment was initiated, with a treatment cycle lasting for 17 days. The specific medication regimen was as follows: progesterone injection (Guangzhou Baiyunshan Mingxing, 20 mg) was administered at a dosage of 40 mg per day via intramuscular injection, dydrogesterone tablets (Dafu Tong, Abbott Laboratories, 10 mg) were taken orally at a dosage of 10 mg, with three times a day. On the sixth day after the start of luteal support treatment, a Day 6 frozen blastocyst that passed chromosomal screening and was deemed available was thawed and transferred. The woman attained a successful pregnancy and delivered a healthy infant in the FET cycle.

### Embryo morphological evaluation

Accordingly, the scoring format was replaced with a numerical shorthand that sequentially represents cell number, stage-specific cell size, and fragmentation (4). For example, ″811″ was defined to embryos with 8 cells and symmetrical stage-specific cell size and less than 10% fragmentation. ″721″ was defined to embryos with 7 cells and unsymmetrical stage-specific cell size and less than 10% fragmentation.

Blastocysts morphology was evaluated according to the scoring system established by Gardner and Schoolcraft (5). Briefly, blastocysts were scored according to their expansive degree and hatching status, as below: (1), an early blastocyst with a blastocoels cavity which is less than half of the embryo volume; (2), a blastocyst with a blastocoels that is half of or larger than half of the embryo volume; (3), a blastocyst with a blastocoel that is full of the whole embryo; (4), an expanded blastocyst with a blastocoel filling the embryo and a thinning zona pellucida; (5), a hatching blastocyst with the trophectoderm beginning to extrude from the zona pellucida; and (6), a hatched blastocyst which has completely escaped from the zona pellucida. In addition, according to the development condition of inner cell mass and trophectoderm, the blastocysts graded as 3–6 was assessed as follows: for the inner cell mass: (A), numerous cells packed tightly; (B), several cells loosely grouped; or (C), only very few cells; for the trophectoderm: (A), large numbers of cells forming a cohesive epithelium; (B), few cells forming a loose epithelium; or (C), very few large cells.

## Clinical results

**Figure 1:**
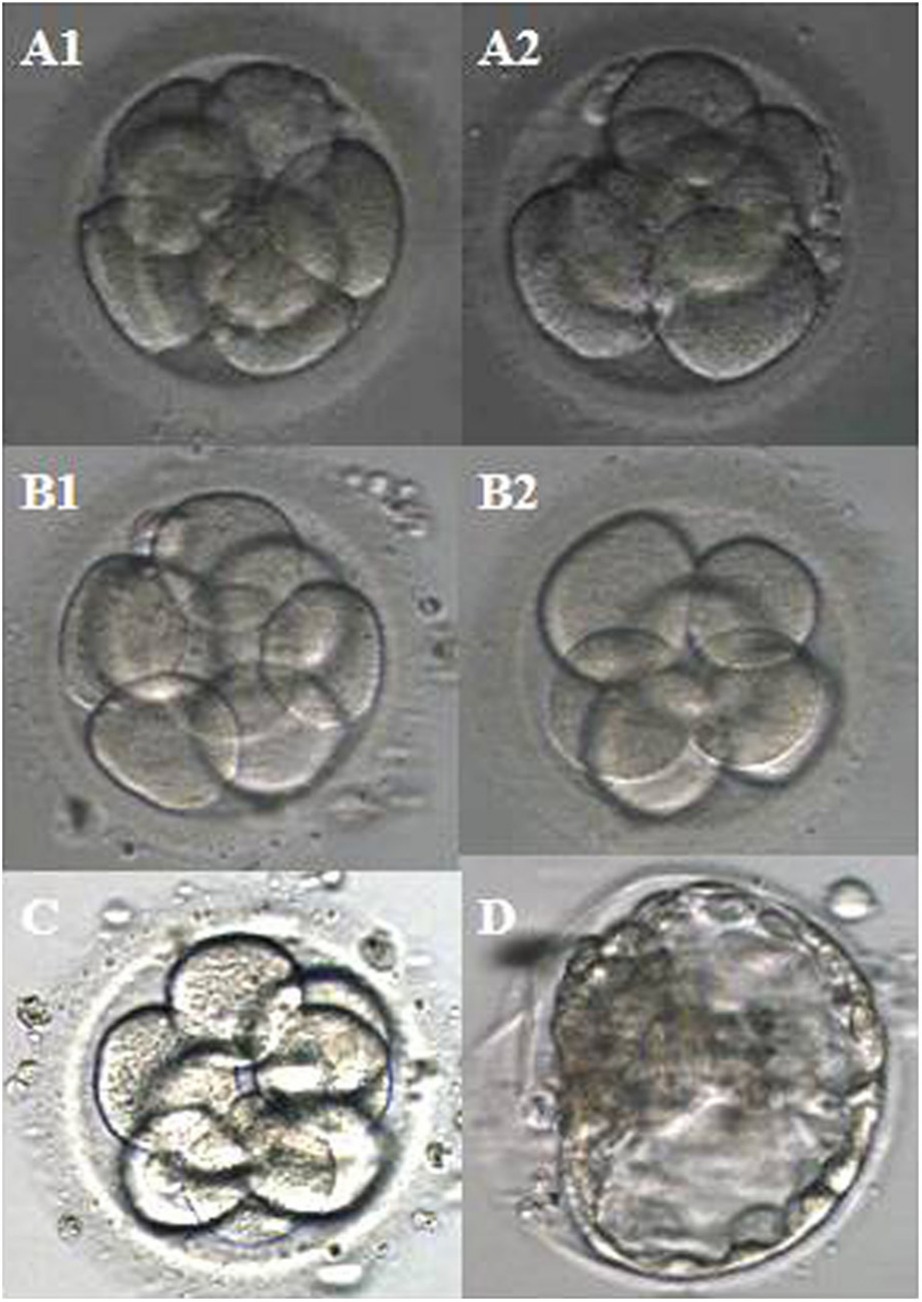
Images of the embryos transplanted per cycle. **(A1**,**A2)** The first cycle; **(B1,B2)** the second cycle; **(C)** the third cycle; **(D)** FET cycle.

This couple underwent a total of three intracytoplasmic sperm injection (ICSI) cycles. All the treatment-related details were comprehensively presented in Table 1, encompassing the quantity of retrieved oocytes, the progress of embryo development, embryo transfer procedures, and the clinical pregnancy outcomes. Meanwhile, Figure 1 showed the images of the embryos transplanted in each cycle.

**Table 1:** Summary of all treatment cycles for the couple.

Cycle	Number of Retrieved oocytes	2PN rate	Day 3 available Embryo rate	Day 3 high-quality Embryo rate	Embryo transfer	Clinical pregnancy Outcome	Abnormal pregnancy outcome
The first cycle	16	25.00% (4/16)	75.00% (3/4)	25.00% (1/4)	811,721	Positive	Early spontaneous abortion (7 weeks)
The second cycle	13	23.08% (3/13)	100% (3/3)	66.67% (2/3)	811,721	Positive	Early spontaneous abortion (12 weeks)
The third cycle	14	42.86% (6/14)	83.33% (5/6)	33.33% (2/6)	811	Positive	Early spontaneous abortion (9 weeks)
FET cycle	–	–	–	–	5BB (46, XN)	Positive	–

2PN, 2 pronucleus; FET, frozen-thawed embryo transfer.

## Discussion

The woman experienced early spontaneous abortions in all three of embryo transfer procedures. The 45, X karyotype identified in chorionic villi of the second cycle may be the underlying etiology. This finding aligned with previous study showing that the proportion of embryonic chromosomal abnormalities in patients with RSA exceeded 40%, which was one of the main factors inducing early abortion (6). Additionally, it has been reported that monosomy X (45, X) was associated with pregnancy loss in humans (7). The more times an abortion occurs, the higher the probability of subsequent abortions (3). Among the known causes of recurrent abortion, embryonic chromosomal abnormalities remain the most common trigger (3). Therefore, for subsequent cycles, PGT-A is recommended to reduce the risk of chromosomally abnormal embryos. When PGT is used to screen out and discard “inferior” embryos, the abortion rate after transplantation decreases significantly (3). Just as in this woman's FET cycle, the transfer of a chromosomally normal blastocyst that had undergone PGT screening resulted in a successful pregnancy and the live birth of a healthy infant, thereby confirming that embryonic chromosomal abnormalities were indeed the primary cause of her three early spontaneous abortions.

A recent study comprehensively analyzed the risk factors for spontaneous abortion after successful pregnancy in patients who underwent laparoscopic surgery for uterine fibroids. The results showed that patients who have undergone laparoscopic surgery for uterine fibroids faced a high risk of spontaneous abortion after successful pregnancy, with age >35 years, uterine fibroid duration >3 years, abnormal uterine cavity morphology after surgery, history of abortion, and fallopian tube obstruction being the risk factors (8). Based on the above-mentioned research, the woman has also undergone myomectomy, there were other relevant factors (except for embryonic chromosomal abnormalities) that may contribute to spontaneous abortion, which warranted further analysis. Her history of uterine fibroids spans less than 1 year and her uterine cavity morphology is normal, thus ruling out the risk factors of uterine fibroid duration >3 years and abnormal uterine cavity structure. Additionally, since she underwent embryo transfer after bilateral salpingectomy, the risk factor of fallopian tube obstruction is also excluded. The patient was 34 years old during her first treatment cycle and over 35 when initiating the second cycle. This age transition may contribute to her risk of spontaneous abortion, though it is unlikely to be the primary risk factor. Evidence for this lied in her experience with a FET cycle: despite being over 35 at the time, she achieved a successful pregnancy and delivered a healthy infant after transferring a chromosomally normal blastocyst. This further confirmed that embryonic chromosomal abnormalities were the main cause of her recurrent spontaneous abortions.

There was evidence indicating that obesity increased the risk of abortion in assisted reproduction (9). Furthermore, an increasing BMI has also been associated with a heightened risk of euploid abortion (10–12). Stanekova et al. have also demonstrated that a high BMI significantly increased the risk of abortion, independent of maternal age and embryo health status (13). The woman's BMI was 22 kg/m^2^, which falls within the normal range. Therefore, her BMI can be ruled out as a high-risk factor for RSA.

Chronic endometritis (CE) is a form of chronic pelvic inflammatory disease linked to infertility, RSA, and other related conditions (14). As a specific type of endometritis, it exerts a notable impact on woman fertility despite presenting with mild clinical symptoms. In patients with RSA, the incidence of CE varies, ranging from 10% to 60% (15). The woman had no evidence of chronic endometritis; thus, her RSAs were not attributable to this factor.

In general, mycoplasma does not induce local infectious symptoms. However, when exposed to certain triggering factors, it can cause retrograde infection, leading to cervicitis, endometritis, urethritis, and salpingitis in women. These conditions directly jeopardize the normal development of early embryos, potentially resulting in abortion (16). The woman showed no evidence of mycoplasma infection or other complications arising therefrom; therefore, this risk factor has also been ruled out as a cause of her RSAs.

While RSA has long been linked to maternal factors, research increasingly highlights the role of paternal contributions—specifically, sperm DNA integrity—in pregnancy success (17). A recent study demonstrated that the integrity of sperm chromatin structure was correlated with RSA (17), aligning with previous research that has also established an association between sperm DNA fragmentation index and RSA (18). Unfortunately, the male partner did not undergo sperm DNA fragmentation testing, so whether this fragmentation rate constituted a high-risk factor for RSA cannot be addressed.

Fetal development is influenced not only by genetics but also by external factors. Ye et al.'s multivariate analysis identified physical labor, exposure to toxic decoration materials, and PM pollution as independent risk factors for RSA (19). The couple denied heavy physical labor, exposure to toxic materials, or air pollution; thus, these external factors were not the cause of their RSAs.

In conclusion, early RSA following assisted reproductive technology (ART) exacerbates both the psychological distress and economic burden of the couple. Clinicians and embryologists conducted a comprehensive analysis of the couple's treatment course to identify the etiological factors underlying RSA. The final analysis revealed that embryonic chromosomal abnormalities constitute the most probable high-risk factor. Consequently, therapeutic strategies were promptly adjusted, with the integration of PGT-A to select chromosomally normal embryos for transfer, thereby achieving successful pregnancy and healthy live birth. This case report provides important clinical references for the assisted reproductive treatment of patients with RSA despite normal chromosomes.

## Data Availability

The original contributions presented in the study are included in the article/Supplementary Material, further inquiries can be directed to the corresponding author.
